# A balanced communication-avoiding support vector machine decision tree method for smart intrusion detection systems

**DOI:** 10.1038/s41598-023-36304-z

**Published:** 2023-06-05

**Authors:** Abdullah Al-Saleh

**Affiliations:** 1 Department of Information Engineering, Florence University, Florence, Italy; 2 Department of Computer Engineering, College of Computer and Information Sciences, Majmaah University, Majmaah, Saudi Arabia

**Keywords:** Engineering, Mathematics and computing, Information theory and computation

## Abstract

The Internet of Things field has created many challenges for network architectures. Ensuring cyberspace security is the primary goal of intrusion detection systems (IDSs). Due to the increases in the number and types of attacks, researchers have sought to improve intrusion detection systems by efficiently protecting the data and devices connected in cyberspace. IDS performance is essentially tied to the amount of data, data dimensionality, and security features. This paper proposes a novel IDS model to improve computational complexity by providing accurate detection in less processing time than other related works. The Gini index method is used to compute the impurity of the security features and refine the selection process. A balanced communication-avoiding support vector machine decision tree method is performed to enhance intrusion detection accuracy. The evaluation is conducted using the UNSW-NB 15 dataset, which is a real dataset and is available publicly. The proposed model achieves high attack detection performance, with an accuracy of approximately 98.5%.

## Introduction

The Internet of Things (IoT) is widely used in our daily lives. Electronic devices had to be connected to the Internet to support monitoring and management. Artificial intelligence (AI) algorithms have added great opportunities to distributed intelligence systems. AI methods form intelligent decision makers and reduce the centralization of decisions, which require considerable time consumption. Nevertheless, the complexity of distributed intelligent systems is continuously increasing^1^. This complexity is revealed in terms of the massive amount of data, the nature of data, the size of datasets, and smart algorithms^2^. These challenges constitute an ideal environment for cyberattacks.

Intrusion Detection Systems (IDSs) have always sought to follow the increase in system complexity. IDS systems aim to protect both physical devices and user data. Therefore, cybersecurity is the key to the success of cloud services. Traditional methods using firewalls, user authentication, and encryption are insufficient to secure devices in cyberspace. This insufficiency is due to the new intrusion detection that is increasing rapidly^3,4^. IDSs have attempted to detect recent attacks such as phishing, denial of service, malware, etc. attacks. The novel IDS seeks to recognize a new attack according to the behavior of the network. Based on the AI algorithm, an IDS classifies whether the network behavior is normal or abnormal.

Machine Learning (ML) approaches have become a vital need for intrusion detection systems. These approaches could achieve accurate classification of network behavior to prevent cyberattacks. Many ML methods such as Support Vector Machines (SVMs), the k-Nearest Neighbors (k-NN), Logistic Regressions (LRs), Decision Trees (DTs), and Naïve Bayes (NB) are used by IDSs to detect intrusions^5–7^. Other methods being used to enhance the attack detection accuracy. All these methods still suffer from many dimensions or features of the data and the massive flow traffic of data. These challenges lead to the complexity of the processing and require considerable time. Therefore, providing a reliable IDS is the main goal in the cybersecurity field.

As a result, intrusion detection systems face the following challenges: (1) multiple natures, dimensionality, and features of data; (2) High data traffic flow; (3) Computational complexity; and (4) Requires considerable time. In light of this introduction, this paper seeks to provide a more accurate intrusion detection system based on the Balanced Communication-Avoiding Support Vector Machine Decision Tree (BCA-SVMDT) method. The proposed aim is to support the complexity by providing accurate detection in less processing time than other related works. The goals are as follows:

1. Model an intrusion detection system based on BCA-SVMDT to efficiently detect cyberspace attacks.
2. Verify the performance of the proposed model according to the accuracy, precision, recall, and F-score.
3. Compare the proposed model with intrusion detection systems based on traditional machine learning methods.

The remainder of this paper is organized as follows. Related works are cited and discussed in section two. Section three describes the proposed intrusion detection system preformed according to the BCA-SVM and DT methods. Experiments and findings are highlighted in section four. Finally, the conclusion and future work are presented in the last section.

## Related works

Intrusion detection systems seek to avoid network attacks. These attacks can be categorized into four essential types:

1. The attacker overloads many resources (memory, network interface, services, etc.). This type of attack is named the Denial of Service (DoS) attack.
2. The attacker attempts to use the system as a normal user. This type of attack is called the Remote-to-Local (R2L) attack.
3. The attacker logs into the system like a normal user and then attempts to change administrator terms. This type of attack is named the User-to-Root (U2R) attack.
4. The attacker tries to scan the network traffic to find useful information to remote access computers. This type of attack is called the probe attack.

In this section, we focus on SVM-based IDS methods proposed in the literature.

Wang et al.^8^ attempted to detect intrusions using a smaller dataset provided by the primary training data. The authors perform three steps to ensure the detection of intrusions as follows: (1) extract the detection models from the dataset, (2) Analyze the training audit data, and (3) Detect network anomalies. The first step is ensured based on the exemplar extraction method. The second step used affinity propagation and K-means clustering. The third step applied Principal Component Analysis (PCA), a k-NN, and an SVM to detect abnormal network behavior. The Knowledge Discovery and Data Mining Tools Competition (KDD Cup) dataset and real HyperText Transfer Protocol (HTTP) traffic are employed to evaluate their intrusion detection system.

He et al.^9^ attempted to accelerate detection by using the twin SVM method, which requires less training time than the SVM. The proposed IDS is composed of twin SVM and Radial Basis Function (RBF) kernels. Unfortunately, this method requires considerable prediction time. The authors evaluated their IDS on R2L and U2R attacks through the KDD Cup dataset. Lin et al.^10^ aggregated SVM and decision tree classifiers to find significant features related to attack behaviors. The proposed method sought to select decision rules using the KDD Cup dataset and detect predicted attacks.

Shang et al.^11^ combined the SVM classifier and Particle Swarm Optimization (PSO) method. The authors aimed to detect anomalies using one class of samples trained by the PSO method. The evaluation is performed on real network traffic data, and the comparisons are limited. Khreich et al.^12^ focused on system calls and traces. The authors aggregated between the frequency and temporal information to be used by the SVM in the training phase. Their IDS is verified according to the Australian Defence Force Academy Linux Dataset (ADFA-LD).

Cid-fuentes et al.^13^ used SVM and decision tree classifiers to improve the accuracy of an IDS. Teng et al. ^14^ built their model on 2-class SVM and decision tree methods. The authors aimed to decrease the overhead and enhance the attack detection rate. Hu et al.^15^ combined the SVM with Adaboost classifiers. The authors used Adaboost because it was an iterative method. Adaboost enhanced the classification performance by learning from the mistakes and weaknesses of classifiers. Hu et al. provided global detection in each node by using Adaboost twice. The first use selected the decision stumps, and the second use improved the online Adaboost.

Aburomman et al.^16^ sought to increase the accuracy of an IDS using a k-NN classifier. Their proposed system used six SVM and six k-NN models in the training phase. The authors performed the PSO and Weighted Majority Algorithm (WMA) methods for the decision phase. Wu et al.^17^ presented an IDS based on deep belief networks and a weighted SVM. The performance of the deep belief network is enhanced by the learning rate method. Then, the SVM is trained using the PSO method. The results lead to an efficient weighted SVM.

Anil et al.^18^ introduced an IDS using the Genetic Algorithm (GA) and entropy function. This method provides a high ability to extract features from the KDD Cup dataset. The authors applied a Self-Organized Feature Map (SOFM) with the SVM to find the similarity between groups in the dataset. The authors showed that their approach achieved a high detection rate with low computation time. Yi et al.^19^ proposed an incremental SVM method to decrease the noise that appeared due to feature differences. A modified kernel function based on the Gaussian function is used with the SVM during the training phase.

Chitrakar et al.^20^ introduced an approach based on an SVM with the half-partition method. The incremental feature of the SVM and the concentric-ring method allowed real-time detection of intrusions. Thaseen et al.^21^ present a method based on multiclass SVM classifiers to detect intrusions. The purpose is to identify several classes according to the network traffic. The authors employed chi-squared filtering instead of the multiclass SVM to enhance the feature selection step. Experimentation is performed using the NSL-KDD dataset and the Libsvm library in the MATLAB environment. The achieved results proved the effectiveness of the proposed method in terms of accuracy and time costs.

Kuang et al.^22^ introduced an IDS model based on the multilayer SVM approach. The model comprises four SVM classifiers and an Improved Chaotic Particle Swarm Optimization (ICPSO) method. The authors sought to detect the four essential types of attacks (R2L, DoS, U2R, and probe). The presented IDS scheme is enhanced by using Principal Component Analysis (PCA) with a SVM to reduce the training time. The experimentation is conducted in the MATLAB environment using the KDD Cup dataset. The findings showed that the method improved the detection accuracy and reduced the processing time in the training and testing phases.

Jaber et al.^23^ sought to model an IDS system using the clustering process. The authors combined the SVM classifier and Fuzzy C-Means (FCM) clustering method to ensure more accurate cloud computing. They conducted experiments using Weka simulation with the NSL-KDD dataset. Safaldin et al.^24^ proposed an IDS scheme using the binary Gray Wolf Optimizer (GWO) as a meta-heuristic method with the SVM. The GWO algorithm to enhance parameters during SVM training. The verification of the proposed model is performed using the NSL-KDD '99 dataset.

Cheng et al.^25^ aggregated the SVM classifier with the bat algorithm to design an IDS model. The bat algorithm is employed in the training phase to find the optimum parameters of the SVM. The KDD Cup '99 dataset is used in the simulation experiments. Raman et al.^26^ conducted an IDS model based on an SVM and a genetic algorithm. A method called the Hypergraph-based Genetic Algorithm (HG-GA) is applied in the selection step to identify the optimum parameters for the SVM classifier. The HG-GA provided the optimal solution and avoided becoming trapped in the local minima. The IDS-based HG-GA SVM is simulated using the NSL-KDD dataset.

Kalita et al.^27^ attempted to handle intrusions using an SVM and Particle Swarm Optimization (PSO). The IDS model based on the SVM classifier achieved higher accuracy when the selected parameters were well chosen. The authors applied a variant of PSO and a multi-PSO algorithm in the selection step to ensure better performance. Li et al.^28^ proposed an IDS model based on the Artificial Bee Colony (ABC) algorithm for feature selection and the SVM classifier. The ABC method is enhanced using honey source coding and the neighborhood search method to retrieve the optimum parameters for the SVM.

Mehmod et al.^29^ sought to improve the selection method before using an SVM classifier to identify attacks. The authors focused on useful features by avoiding noise and redundancy. The selection method is performed by applying the ant colony optimization algorithm on the KDD Cup '99 dataset. Acharya et al.^30^ adopted a general approach-based SVM to design an IDS. Regarding the selection step, the authors proposed an intelligent water drop (IWD) algorithm to select the relevant features for classification. The KDD Cup '99 dataset is used to evaluate the proposed IDS.

Li et al.^31^ stated that the Velocity Adaptive Shuffled Frog Leaping Bat Algorithm (VASFLBA) was an effective method for the selection process. The procedure is based on two adaptative factors to balance global and local search. The Shuffled Frog Leaping Algorithm (SFLA) improved the transfer mechanism. The selected features were trained according to the SVM classifiers on the Industrial Control System (ICS) dataset. Bostani et al.^32^ designed an IDS system based on hybrid feature selection. A Binary Gravitational Search Algorithm (BGSA) and Mutual Information (MI) were used to perform the selection step. Experimentation is conducted using the NSL-KDD dataset.

Kabir et al.^33^ introduced the Least Squares Support Vector Machine (LS-SVM) to build an accurate IDS. The optimum allocation algorithm proceeds to select representative samples. The IDS is tested using the KDD Cup '99 dataset. Saleh et al.^34^ proposed a Hybrid IDS (HIDS) based on multiclass classification. The selection step uses the Naïve Bayes Feature Selection (NBFS) method. It aimed to decrease the dimensionality of sample data. The model rejects outliers utilizing an Optimized Support Vector Machine (OSVM) classifier in training. Then, a Prioritized k-Nearest Neighbors (PKNN) technique is employed to detect attacks. The findings on the KDD Cup '99, NSL-KDD, and Kyoto 2006+ datasets proved the detection accuracy at a low time cost.

Nskh et al.^35^ modeled an IDS based on different SVM kernels. The authors reduced the dimensionality of the dataset by applying Principal Component Analysis (PCA) and adopting the Gaussian radial basis function kernel of the SVM. Wang et al.^36^ focused on the time-consuming drawbacks related to an IDS. The authors introduced a parallel model based on a PCA-SVM implemented in the Spark board. The PCA ensures the training phase, and the SVM is fused through the bagging integration technique.

In light of this brief description of the related works, IDSs still face the following five challenges^37,38^:

1. *Large dataset challenge* A large amount of data in a dataset leads to highly time-consuming training steps. Exemplar extraction methods and clustering methods are proposed to reduce the dataset size without losing relevant information.
2. *Normalization challenge* The quality of data directly influences the accuracy of intrusion detection systems. The normalization method rebuilds data to obtain valuable data and reduces the processing time. Selecting the best normalization method is a crucial step for IDS.
3. *SVM learning method challenge* The SVM, as a supervised learning method, efficiently handles labeled data. Moreover, unlabeled data are found in applications, and the SVM classifier is limited in real cases. Semisupervised methods were proposed in the literature to support both labeled and unlabeled data.
4. *Incremental learning challenge* Since training data are unavailable all time, an IDS becomes unable to detect new attacks. Real-time IDS supporting frequent retraining (incremental learning) is the best solution.
5. *Online learning challenge* As an SVM does not support periodic retraining, the classifier cannot manage the requests of an online intrusion detection system. Some attempts use an online SVM to support online learning demands.

In this paper, the proposed IDS seeks to address the above challenges. The model comprises a selection method and a hybrid classifier based on the Balanced Communication-Avoiding Support Vector Machine Decision Tree (BCA-SVMDT) method. The selection method aims to select the most significant features to be trained. The BCA-SVMDT, which is discussed in the next section, ensures the training phase.

## BCA-SVMDT-based intrusion detection system

**Figure 1:**
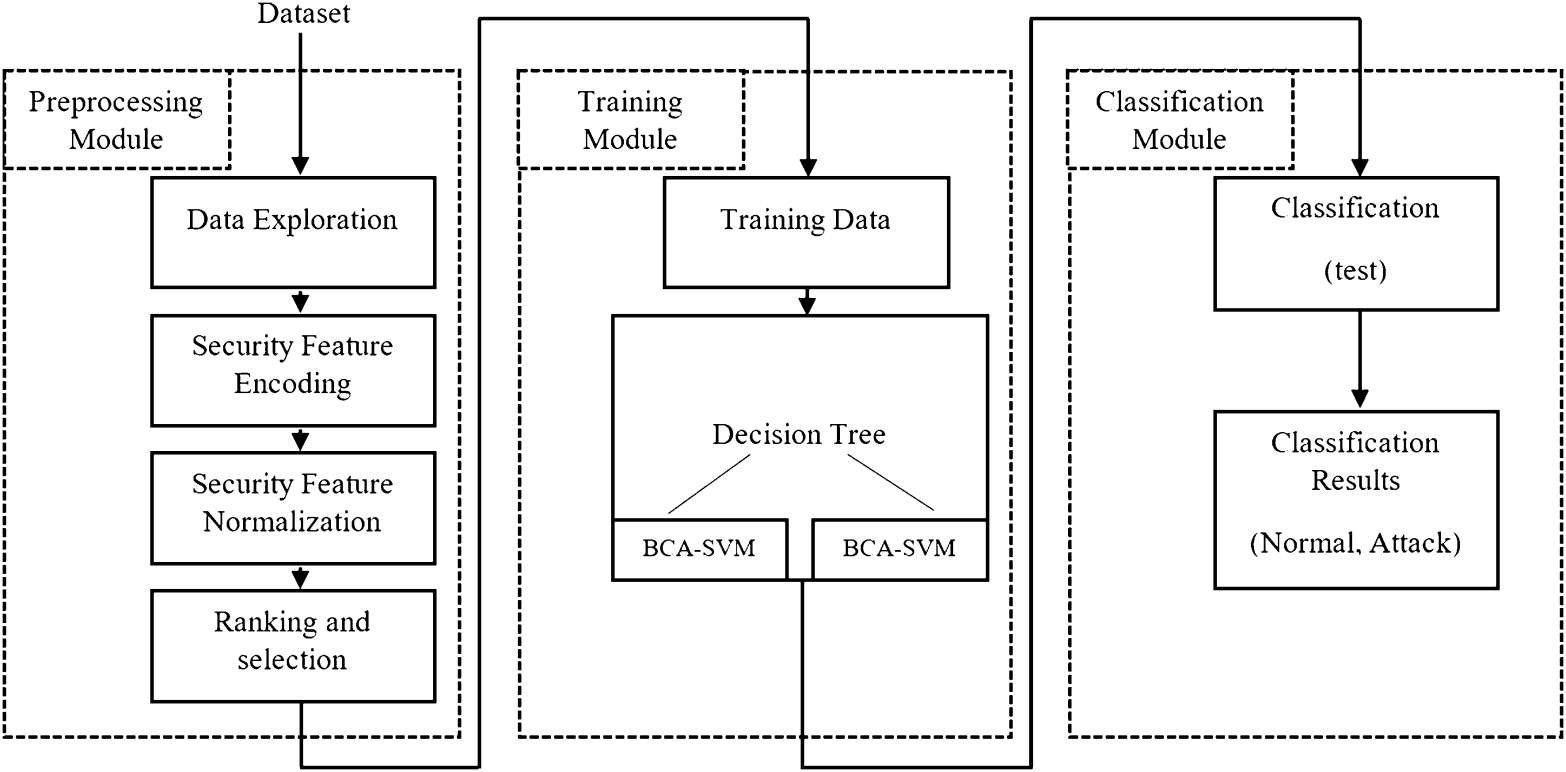
BCA-SVMDT model.

The proposed model is introduced in this section. The IDS model is composed of three main modules, as shown in Fig. 1. The intrusion model is built based on a decision tree; and on a particular node, the BCA-SVM classifier is used. The illustrated model in Fig. 1 is detailed in the following sections.

### Preprocessing module

#### Data exploration

This step is focused on the quality of the data. To ensure the accuracy of the prediction model, data exploration inspects the data to explore its features. The type of data (numerical or categorical) is verified to determine a suitable statistical or prediction model. In our case, the UNSW-NB 15 dataset is used^39^. This dataset is available online and is composed of 175,341 records. The UNSW-NB 15 dataset encompasses 44 features, including normal and attack status. The data exploration process determines three features (proto, state, and service) that are nominal. The other features are defined by numerical values (binary, integer, and floating). Nominal features have to be considered for the next step (security feature encoding) to be transformed from nominal values to numerical values.

#### Security feature encoding

**Figure 2:**
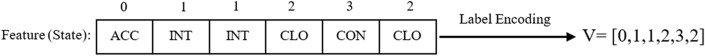
Label encoding method.

This step encodes the nominal values determined by the data exploration step. Nominal features (proto, state, and service) are encoded using the label encoding method. This method did not create additional features like the one hot encoding method. This is why the label encoding method is chosen to transform these three features from nominal values into numerical values. The method labels the same parameter with the same numerical value. The example illustrated in Fig. 2 describes the label encoding method. The security feature encoding step is performed using the LabelEncoder method and sklearn class in Python.

#### Security feature normalization

This step manages data with different scales. It aims to rescale the values of all features according to a zero mean and a unit variation. The normalization process is fundamental in the training phase to provide an accurate classification model. The rescaled value is computed through the following equation.

1 $${D}_{S}=\frac{{D}_{i}-\overline{D}}{\sigma }$$

${D}_{S}$ is the scaled value, ${D}_{i}$ is the original value, $\overline{D }$ is the mean value of the feature, and the standard deviation is represented by $\sigma$. Normalization is performed for every feature that has a different distribution using the sklearn class in Python.

#### Ranking and selection

This step aims to select significant features that support the decision-making process. The Gini index method is applied to ensure feature ranking. It has been employed on Binary attack and benign data, whereas the Gini index works better on multiclass data^40^. The Gini index method is performed as follows: (1) it detects the impurity of the features; (2) it ranks the features based on the Gini impurity, which is defined by the entropy; and (3) it builds the decision tree. The Gini index is computed in every node using Eq. (2).

2 $${G}_{index}(n)=1-\sum_{i=1}^{T}{({P}_{i})}^{2}$$

where n is a node, T is the number of all nodes and ${P}_{i}$ is the probability of a tuple.

The Gini index is applied for all features in the UNSW-NB 15 dataset. Table 1 illustrates the ranking associated with the security features.

**Table 1:** FEATURE IMPORTANCE SCORE.

Security feature	Score
Sttl	0.122
Ct_dst_src_itm	0.080
Ct_state_ttl	0.079
Swin	0.070
Dttl	0.062
Ct_srv_dst	0.040
State	0.040
Ct_dst_sport_itm	0.039
Smean	0.038
Ct_srv_src	0.036
Service	0.034
Rate	0.030
Dload	0.025
Sbytes	0.024
Dmean	0.023
Dwin	0.020
Synack	0.018
Sload	0.017
Ct_src_dport_itm	0.016
Tcprtt	0.016
Sinpkt	0.015
Ct_dst_itm	0.014
Ct_src_itm	0.014
Ackdat	0.012
Proto	0.010
Dur	0.010
Dtcpb	0.008
Trans_depth	0.008
Stcpb	0.007
Dbytes	0.007
Djit	0.006
Sjit	0.005
Dinpkt	0.005
Sloss	0.004
Dpkts	0.004
Ct_flw_http_mthd	0.004
Is_sm_ips_ports	0.004
Dloss	0.004
Spkts	0.003
Response_body_len	0.002
Is_ftp_login	0.001
Ct_ftp_cmd	0.001

The selection of the important security features is made according to the threshold (threshold = 0.023) which is defined through the Tree model. The Threshold value could be changed according to the used dataset. The number of selected features is reduced from 42 to 15 features.

**Figure 3:**
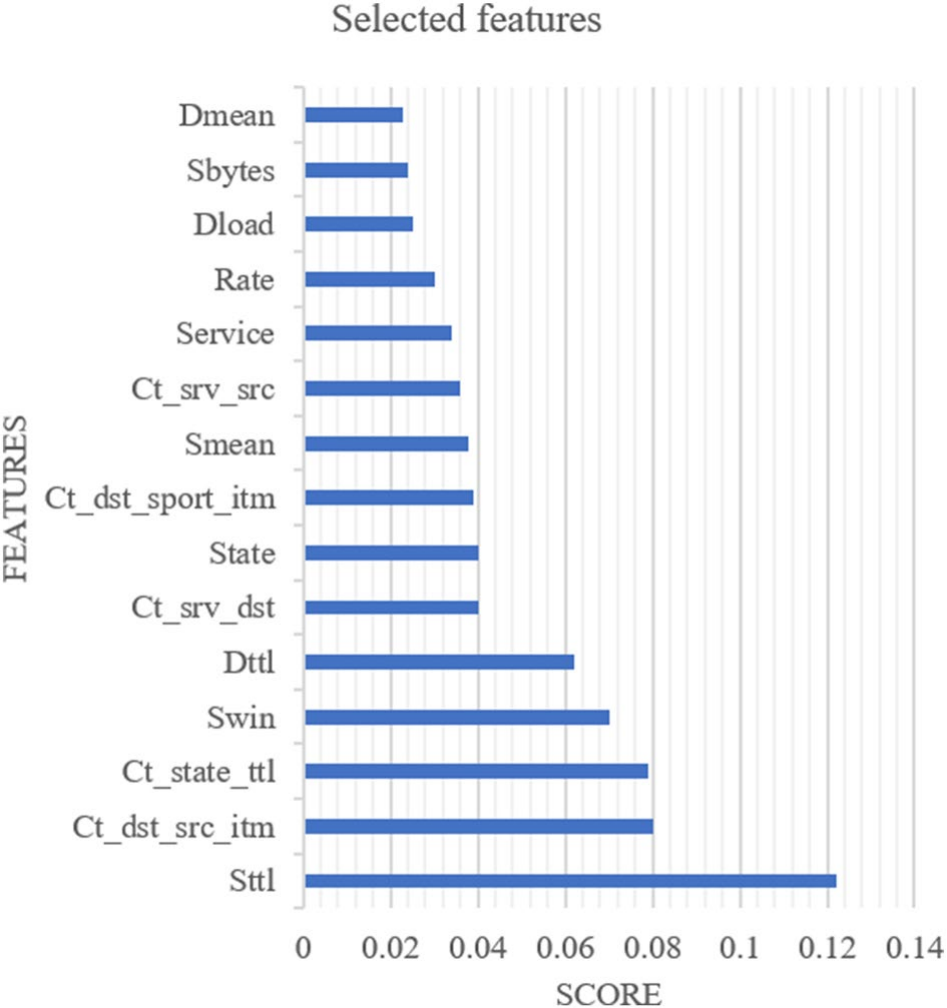
The selected security features based on the threshold.

Figure 3 shows the selected features and their scores. As mentioned above, this step helps to reduce the computational complexity and increase the accuracy of the proposed decision tree-BCA-SVM classification.

### Training module

**Figure 4:**
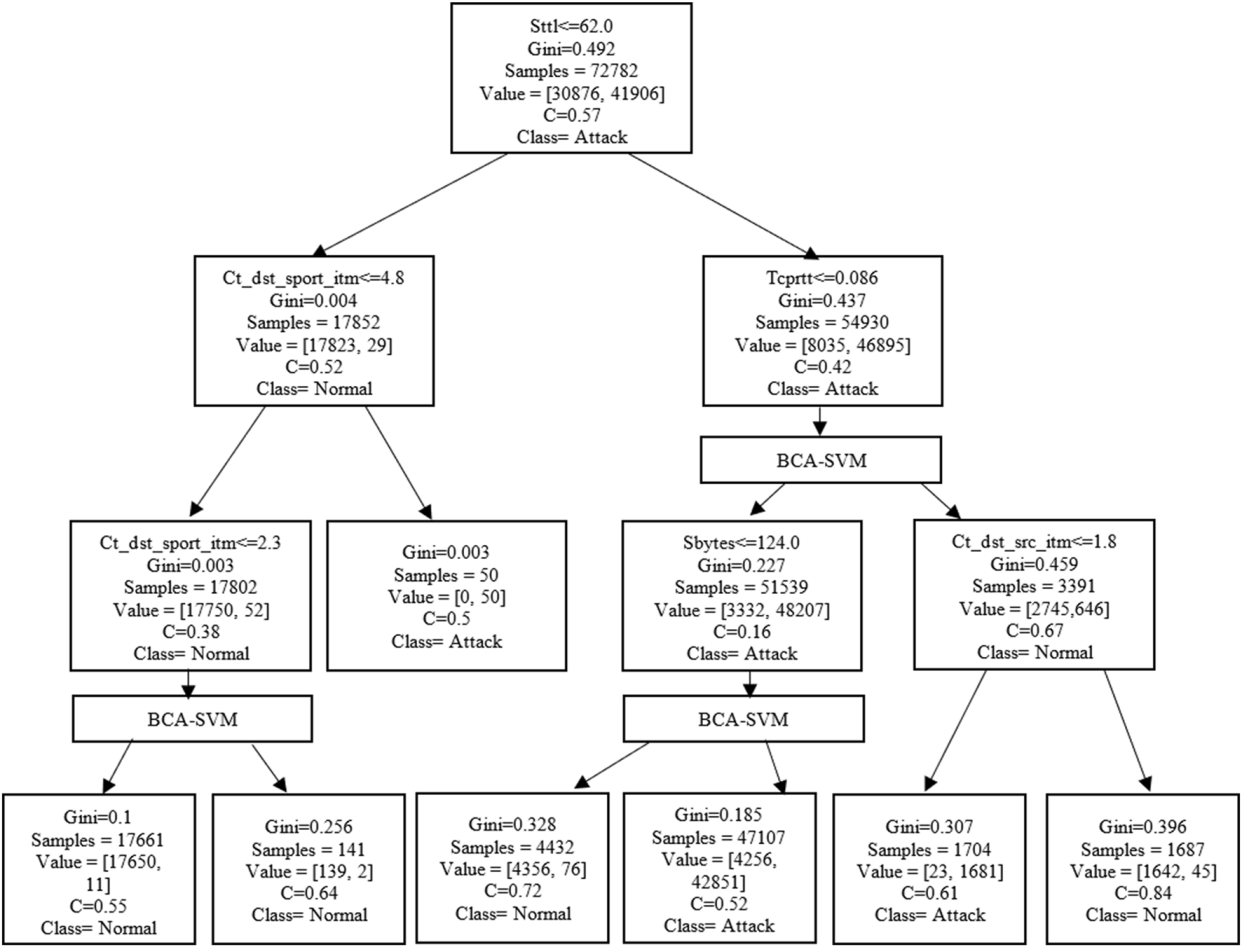
The BCA-SVMDT tree.

The training module is performed based on hybrid BCA-SVM and decision tree methods. The BCA-SVM classifier presents an optimized SVM version and achieves better classification results. Figure 4 illustrates the BCA-SVMDT intrusion detection tree.

The sttl feature chosen by the Gini index method is considered the root node. Branches were added based on the feature name, Gini index, samples, value, closeness measure (c), and class name. This module is performed in the local learning model according to the following steps:

1. Select the SVM kernel functions (radial basis function) with the C regulation parameter and the σ kernel parameter. These parameters are chosen according to the validation results.
2. Train the BCA-SVM classifier to find the decision function f(x).
3. Classify training data into normal class or attack class.
4. Store the classification prediction in the new target.
5. Train the decision tree with training data and a new target.
6. Replace the class with the BCA-SVM when the closeness measure (c) is less than 0.5.
7. Save the tree.

**Figure 5:**
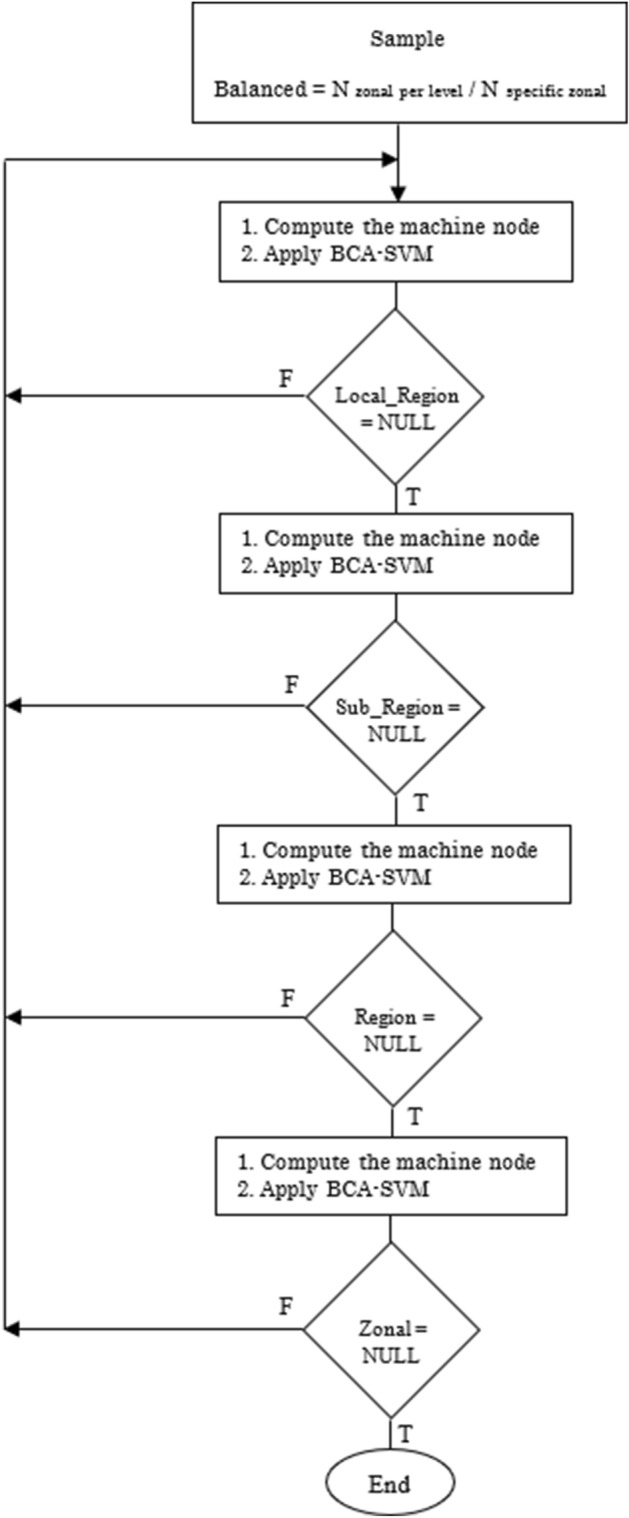
Flowchart of the BCA-SVM classifier.

The BCA-SVM learning step is summarized in Fig. 5.

In the next section, we detail the experiments and the evaluation of the proposed BCA-SVMDT model.

## Experiments

In this section, the proposed BCA-SVMDT intrusion detection system is evaluated using the UNSW-NB 15 dataset. This dataset was created by the Cyber Range Lab of the Australian Center for Cyber Security (ACCS)^37^. As mentioned in section “BCA-SVMDT-based intrusion detection system”, the dataset is composed of 42 features. In our research, only 15 relevant features that are more significant are used.

The training phase aims to build two classes: normal or attack. The nature of the attack is outside the scope of this research. For the training, the proposed model used 120,890 records. For the test phase, 16,607 records are covered. The experimentation is conducted in Python 3.8 running on a computer with a core i7 CPU and 8 GB RAM.

The evaluation is performed using four metrics: the accuracy, precision, recall, and F-score. These metrics are important for comparing the proposed IDS and some traditional Machine Learning (ML) models. The evaluation metrics are computed based on the following values:

- TP (True Positives) denotes the number of correctly detected intrusions.
- TN (True Negatives) denotes the number of correctly detected normal network statuses (nonintrusions).
- FP (False Positives) denotes the number of normal statuses detected as intrusions.
- FN (False Negatives) denotes the number of intrusions detected as normal statuses.
- The accuracy reflects the rate of correct predictions. It is computed through Eq. (3).
  3 $$Accuracy= \frac{TP+TN}{TP+TN+FP+FN}$$

The precision represents the rate of correct detections belonging to the right class. It is represented using Eq. (4).

4 $$Precision= \frac{TP}{TP+FP}$$

The recall represents the number of correct detections divided by all intrusion cases in the dataset. Equation 5 shows the recall formula.

5 $$Recall= \frac{TP}{TP+FN}$$

The F-score metric balances the precision and recall. It is described by Eq. (6).

6 $$Fscore=2\times \frac{Recall \times Precision}{Recall+Precision}$$

Table 2 illustrates the results of the experiments in the testing phase. The average accuracy is approximately 98.5%.

**Table 2:** Results of the BCA-SVMDT model for intrusion detection.

Class	Accuracy (%)	Precision (%)	Recall (%)	F-score (%)
Normal	98.4	97.8	96.2	97
Attack	98.6	95.6	96.6	96
Average	98.5	96.7	96.4	96.5

**Figure 6:**
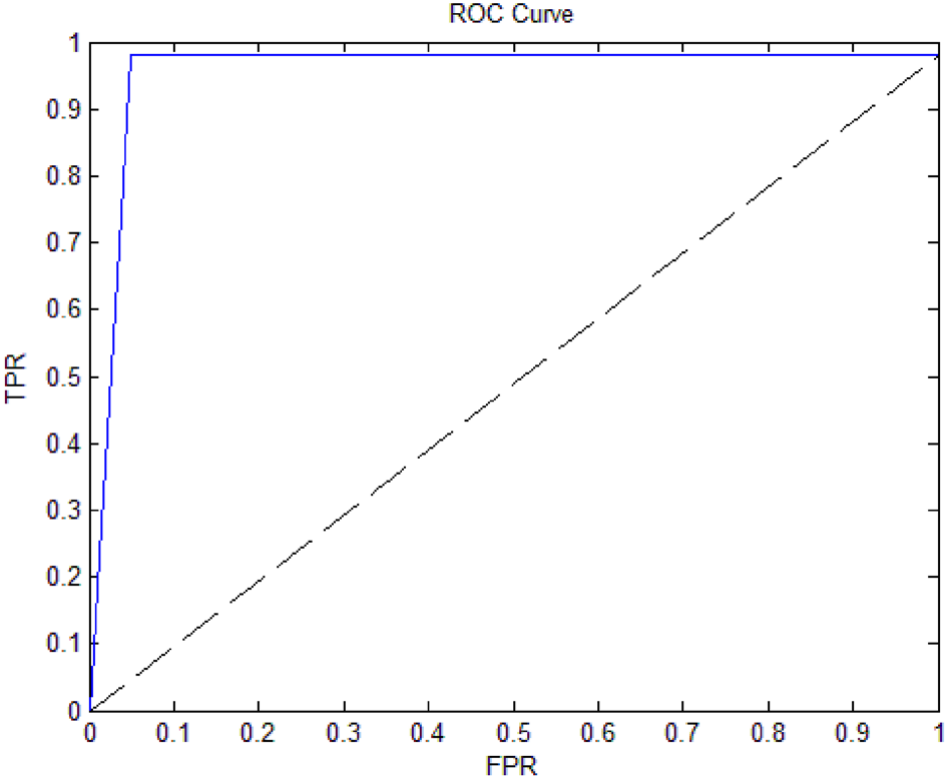
The receiver operating curve of the BCA-SVMDT model.

The proposed model is also evaluated according to the Receiver Operating Curve (ROC). The ROC curve gives an idea about the performance of the BCA-SVMDT model and the distance between the two classes: normal and attack. The ROC curve is defined by Eq. 7.

7 $$\left\{ {\begin{array}{*{20}l} {ROC = f\left( {TPR,FPR} \right)} \hfill \\ {FPR = \frac{{FP}}{{FP + TN}}} \hfill \\ \end{array} } \right.$$

where TPR is the True Positive Rate, and FPR is the False Positive Rate. The TPR value equals the Recall value. The ROC curve is drawn in Fig. 6. In Fig. 6, the prediction model is accurate on the higher Area Under the Curve (AUC), which is approximately 0.98.

**Figure 7:**
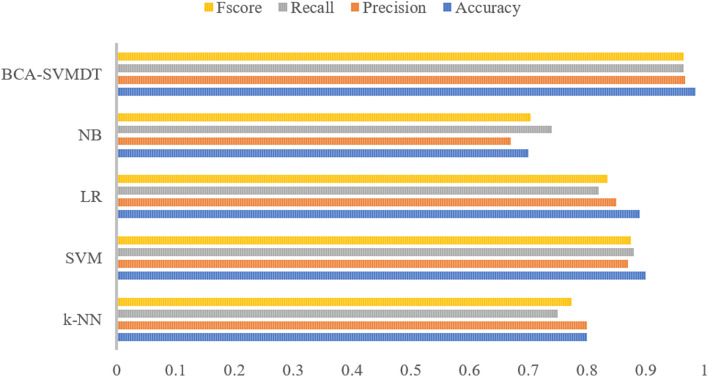
Comparison results between the BCA-SVMDT model and traditional ML models.

Traditional models based on machine learning methods such as the SVM, k-Nearest Neighbors (k-NN), Logistic Regression (LR), and Naïve Bayes (NB) are applied to the same dataset to assess the benefits of the proposed model in depth. Figure 7 illustrates the comparison between the proposed BCA-SVMDT and the other ML methods according to the accuracy, precision, recall, and F-score metrics. The results prove that the BCA-SVMDT method for intrusion detection achieves the best performance.

The proposed IDS model decreases the computational complexity using the ranked security features for the selection approach. Therefore, the processing time and overfitting are improved.

## Conclusion

Protecting networks from intrusions and attacks is a great challenge for cyberspace. In this paper, an attempt to provide an accurate IDS based on a hybrid approach is presented. A novel intelligent system called BCA-SVMDT composed of a decision tree and a balanced communication-avoiding support vector machine classifier is proposed to optimize the training phase. In the preprocessing module, the data are rescaled and encoded. The Gini index method is performed to compute the impurity of the security features. Our model reached a high accuracy of approximately 98.5%, a precision of approximately 96.7%, a recall of approximately 96.4%, and an F-score of approximately 96.5%. Furthermore, this paper will be a solid key to predicting the nature of attacks in future work. An enhancement of the IDS model is required by adding a filtering step to enhance the prediction and support the classification of five classes, including normal status and types of attacks.

## Data Availability

The datasets generated and/or analyzed during the current study are available in the Kaggle repository, https://www.kaggle.com/datasets/dhoogla/unswnb15.

## References

[1] Wang G (2021). Comparative study on different neural networks for network security situation prediction. Secur. Priv..

[2] Hesselman C, Grosso P, Holz R (2020). A responsible internet to increase trust in the digital world. J. Netw. Syst. Manage.

[3] Bhuyan MH, Bhattacharyya DK, Kalita JK (2014). Network anomaly detection: Methods, systems and tools. IEEE Commun. Surv. Tutor..

[4] Tapiador JE, Orfila A, Ribagorda A, Ramos B (2015). Key-recovery attacks on KIDS, a keyed anomaly detection system. IEEE Trans. Dependable Secure Comput..

[5] Buczak AL, Guven E (2016). A survey of data mining and machine learning methods for cyber security intrusion detection. IEEE Commun. Surv. Tutor..

[6] Mishra P, Varadharajan V, Tupakula U, Pilli ES (2019). A detailed investigation and analysis of using machine learning techniques for intrusion detection. IEEE Commun. Surv. Tutor..

[7] Lopez-Martin M, Carro B, Sanchez-Esguevillas A (2020). Application of deep reinforcement learning to intrusion detection for supervised problems. Expert Syst. Appl..

[8] Wang W, Liu J, Pitsilis G, Zhang X (2018). Abstracting massive data for lightweight intrusion detection in computer networks. Inf. Sci..

[9] He J, Zheng S-H (2014). Intrusion detection model with twin support vector machines. J. Shanghai Jiaotong Univ. Sci..

[10] Lin S, Ying K, Lee C, Lee Z (2012). An intelligent algorithm with feature selection and decision rules applied to anomaly intrusion detection. Appl. Soft Comput..

[11] Shang, W., Li, L., Wan, M. and Zeng, P. Industrial communication intrusion detection algorithm based on improved one-class SVM. *2015 World Congress on Industrial Control Systems Security (WCICSS)*, London, 21–25, (2015). 10.1109/WCICSS.2015.7420317

[12] Khreich W, Khosravifar B, Hamou-Lhadj A, Talhi C (2017). An anomaly detection system based on variable N-gram features and one-class SVM. Inf. Softw. Technol..

[13] Álvarez J, Szabo C, Falkner K (2020). Adaptive performance anomaly detection in distributed systems using online SVMs. IEEE Trans. Dependable Secure Comput..

[14] Teng S, Wu N, Zhu H, Teng L, Zhang W (2018). SVM-DT-based adaptive and collaborative intrusion detection. IEEE/CAA J. Automatica Sinica.

[15] Hu W, Gao J, Wang Y, Wu O, Maybank S (2014). Online adaboost-based parameterized methods for dynamic distributed network intrusion detection. IEEE Transact. Cybern..

[16] Aburomman AA, Ibne Reaz MB (2016). A novel SVM-kNN-PSO ensemble method for intrusion detection system. Appl. Soft Comput..

[17] Wu Y, Lee W, Xu Z, Ni M (2020). Large-scale and robust intrusion detection model combining improved deep belief network with feature-weighted SVM. IEEE Access.

[18] Anil, S. and Remya, R. A hybrid method based on genetic algorithm, self-organised feature map, and support vector machine for better network anomaly detection. *2013 Fourth International Conference on Computing, Communications and Networking Technologies (ICCCNT)*, Tiruchengode, India, 1–5, (2013). 10.1109/ICCCNT.2013.6726604

[19] Yi Y, Wu J, Xu W (2011). Incremental SVM based on reserved set for network intrusion detection. Expert Syst. Appl..

[20] Chitrakar R, Huang C (2014). Selection of candidate support vectors in incremental SVM for network intrusion detection. Comput. Secur..

[21] Sumaiya Thaseen I, Aswani Kumar C (2017). Intrusion detection model using fusion of chi-square feature selection and multi class SVM. J. King Saud Univ. Comput. Inform. Sci..

[22] Kuang F, Zhang S, Jin Z (2015). A novel SVM by combining kernel principal component analysis and improved chaotic particle swarm optimization for intrusion detection. Soft Comput..

[23] Jaber AN, Rehman SU (2020). FCM–SVM based intrusion detection system for cloud computing environment. Cluster Comput..

[24] Safaldin M, Otair M, Abualigah L (2021). Improved binary gray wolf optimizer and SVM for intrusion detection system in wireless sensor networks. J. Ambient Intell. Human Comput..

[25] Cheng, C., Bao, L., Bao, C. Network intrusion detection with bat algorithm for synchronization of feature selection and support vector machines. In: Cheng, L., Liu, Q., Ronzhin, A. (eds) Advances in Neural Networks – ISNN 2016. ISNN 2016. Lecture Notes in Computer Science(), vol 9719. (Springer, Cham, 2016) 10.1007/978-3-319-40663-3_46

[26] Gauthama Raman M, Somu N, Kirthivasan K, Liscano R, Shankar Sriram V (2017). An efficient intrusion detection system based on hypergraph–genetic algorithm for parameter optimization and feature selection in support vector machine. Knowl.-Based Syst..

[27] Kalita, D. J., Singh, V. P., Kumar, V. SVM hyper-parameters optimization using multi-PSO for intrusion detection. Shukla, R., Agrawal, J., Sharma, S., Chaudhari, N., Shukla, K. (eds) *Social Networking and Computational Intelligence.* Lecture Notes in Networks and Systems, **100**. (Springer, Singapore, 2020). 10.1007/978-981-15-2071-6_19

[28] Li, L., Zhang, S., Zhang, Y., Chang, L. and Gu, T. The intrusion detection model based on parallel multi - artificial bee colony and support vector machine. *2019 Eleventh International Conference on Advanced Computational Intelligence (ICACI)*, Guilin, China, 308–313, (2019). 10.1109/ICACI.2019.8778482

[29] Mehmod, T., & Rais, H. B. M. Ant colony optimization and feature selection for intrusion detection. Soh, P., Woo, W., Sulaiman, H., Othman, M., Saat, M. (eds) *Advances in Machine Learning and Signal Processing*. Lecture notes in electrical engineering, **387**, (Springer, Cham, 2016). 10.1007/978-3-319-32213-1_27

[30] Acharya N, Singh S (2018). An IWD-based feature selection method for intrusion detection system. Soft Comput..

[31] Li, J., Wang, H. and Yan, B. Application of velocity adaptive shuffled frog leaping bat algorithm in ICS intrusion detection. *2017 29th Chinese Control And Decision Conference (CCDC)*, Chongqing, 3630–3635, (2017). 10.1109/CCDC.2017.7979135

[32] Bostani H, Sheikhan M (2017). Hybrid of binary gravitational search algorithm and mutual information for feature selection in intrusion detection systems. Soft. Comput..

[33] Kabir E, Hu J, Wang H, Zhuo G (2018). A novel statistical technique for intrusion detection systems. Futur. Gener. Comput. Syst..

[34] Saleh AI, Talaat FM, Labib LM (2019). A hybrid intrusion detection system (HIDS) based on prioritized k-nearest neighbors and optimized SVM classifiers. Artif. Intell. Rev..

[35] Nskh, P., Varma, M. N. and Naik, R. R. Principle component analysis based intrusion detection system using support vector machine. *2016 IEEE International Conference on Recent Trends in Electronics, Information & Communication Technology (RTEICT)*, Bangalore, India, 1344–1350, (2016). 10.1109/RTEICT.2016.7808050

[36] Wang, H., Xiao, Y. and Long, Y. Research of intrusion detection algorithm based on parallel SVM on spark. *2017 7th IEEE International Conference on Electronics Information and Emergency Communication (ICEIEC)*, Macau, China, 153–156, (2017) 10.1109/ICEIEC.2017.8076533

[37] Khraisat A, Gondal I, Vamplew P (2019). Survey of intrusion detection systems: Techniques, datasets and challenges. Cybersecur.

[38] Meng W, Tischhauser EW, Wang Q, Wang Y, Han J (2018). When intrusion detection meets blockchain technology: A review. IEEE Access.

[39] Rajagopal, S., Hareesha, K. S., Kundapur, P. P. Feature relevance analysis and feature reduction of UNSW NB-15 using neural networks on MAMLS. Pati, B., Panigrahi, C., Buyya, R., Li, KC. (eds) advanced computing and intelligent engineering. *Advances in Intelligent Systems and Computing*, **1082**. (Springer, Singapore, 2020). 10.1007/978-981-15-1081-6_27

[40] Test, E., Zigic, L. and Kecman, V. Feature ranking using Gini index, scatter ratios, and nonlinear SVM RFE. *2013 Proceedings of IEEE Southeastcon, Jacksonville*, FL, USA, 1–5, (2013). 10.1109/SECON.2013.6567380

